# RACIAL DIFFERENCES IN LONGITUDINAL TRAJECTORIES OF SOCIAL ISOLATION AND LONELINESS

**DOI:** 10.1093/geroni/igad104.1393

**Published:** 2023-12-21

**Authors:** Harry Taylor

**Affiliations:** University of Toronto, Toronto, Ontario, Canada

## Abstract

The purpose of this study was to determine if there are racial differences in the longitudinal trajectories of social isolation and loneliness among older adults. Data come from the Health and Retirement Study Leave Behind Questionnaire, waves 2006-2016. Social isolation was operationalized as a social network index and loneliness was operationalized by the UCLA-3 item loneliness scale. Race was operationalized by White, Black, and Hispanic. I also adjusted for age, gender, education, household income, and employment status. Racial differences in longitudinal trajectories were determined by multilevel models with survey weights included by the HRS. In unadjusted models, I found Hispanic older adults were less likely to be socially isolated compared to White and Black older adults; furthermore, White older adults were less likely to be lonely compared to Black and Hispanic older adults. In fully adjusted models, White older adults had greater social isolation in comparison to Black and Hispanic older adults, and Black older adults had greater isolation compared to Hispanic older adults. For loneliness, Black older adults were found to be lonelier compared to Hispanic older adults, but not White older adults. This study is one of the first to examine racial differences in longitudinal trajectories of social isolation and loneliness. I find race is a salient factor that may influence objective isolation and feelings of loneliness among older adults. Given the established relationship between isolation, loneliness, and health, these findings are useful for identifying potential racial groups at higher risk for these conditions and informing future interventions.

